# Wild *Nepeta sibirica* L. from Eastern Kazakhstan: Botanical Characterization, Essential-Oil Composition, Chemogeographic Context, and Preliminary Bioactivity

**DOI:** 10.3390/plants15192992

**Published:** 2026-09-30

**Authors:** Anar Myrzagaliyeva, Milena Rašeta, Talant Samarkhanov, Yerlan Suleimen, Zharkyn Ibatayev, Moldir Sharipova, Nargiza Adilova, Gulnur Mamytbekova, Yusufjon Gafforov

**Affiliations:** 1 Research Laboratory “NatureLaB”, Astana International University, Astana 010000, Kazakhstan; an.myrzagaliyeva@gmail.com (A.M.); talant.68@mail.ru (T.S.); 2 Department of Chemistry, Biochemistry and Environmental Protection, Faculty of Sciences, University of Novi Sad, 21000 Novi Sad, Serbia; 3 Department of Chemical Engineering and Efficient Use of Natural Resources, Kulazhanov Kazakh University of Technology and Business, Astana 010000, Kazakhstan; syerlan75@yandex.ru (Y.S.); gulnur4284@mail.ru (G.M.); 4 Department of Physics and Chemistry, Saken Seifullin Kazakh Agrotechnical University, Astana 010000, Kazakhstan; zh.ibatayev@kazatu.kz; 5 Department of Family Medicine No. 2 Clinical Pharmacology, National Medical Center, Tashkent State Medical University, Tashkent 100109, Uzbekistan; adilova.nargiza82@gmail.com; 6 Department of Botany and Genetics, Faculty of Biology and Ecology, National University of Uzbekistan, Tashkent 100174, Uzbekistan; y.gafforov@newuu.uz; 7 Department of Medicine, Faculty of Medicine, Alfraganus University, Tashkent 100190, Uzbekistan; 8 Faculty of Chemical Technology, Fergana State Technical University, Fergana 150100, Uzbekistan

**Keywords:** Altai, *Artemia salina*, chemogeography, essential oil, GBIF, GC–MS, Kazakhstan, Lamiaceae, *Nepeta*, nepetalactone, stereochemistry

## Abstract

*Nepeta sibirica* L. is a medicinal and aromatic species of Lamiaceae whose volatile chemistry varies across its Eurasian range. In this study, we integrated voucher-anchored botanical characterization, GBIF-supported distributional context, essential-oil composition, chemogeographic comparison, and preliminary bioactivity assessment of a wild population from the Altai–Tarbagatai Ridge, Eastern Kazakhstan. Hydrodistillation yielded 0.3% (*w*/*w*) essential oil. GC–MS analysis of the essential oil detected and tentatively identified 62 volatile constituents, accounting for 95.5% of the total composition. A dominant nepetalactone peak represented 70.6% of the oil and was tentatively assigned as *cis–trans*-nepetalactone based on electron-ionization mass-spectral and retention-index data obtained on an achiral column. Germacrene D (6.5%) and *cis*-β-ocimene (2.8%) were the principal secondary constituents. Chemogeographic comparison indicated similarity to previously reported nepetalactone-rich material from the Altai region. The essential oil exhibited limited DPPH radical-scavenging activity, reaching a maximum inhibition of 12.88 ± 0.56% at 24.19 µg/mL; an IC_50_ could not be determined within the tested concentration range. In the *Artemia salina* assay, the essential oil caused 100% mortality at all tested concentrations (0.01–0.10 mg/mL) after 24 h, precluding LC_50_ estimation within the tested range. These findings document a nepetalactone-dominant essential-oil profile in a voucher-confirmed wild population of *N. sibirica* from Eastern Kazakhstan and provide preliminary quantitative data on its DPPH radical-scavenging activity and *A. salina* lethality.

## 1. Introduction

Medicinal and aromatic plants are important sources of specialised metabolites and essential oils with pharmaceutical, cosmetic, agricultural, and ecological relevance. Their volatile profiles can vary with taxon, geography, habitat, phenological stage, plant organ, extraction procedure, and analytical conditions. Population-based studies are therefore particularly valuable for interpreting chemically distinctive wild resources and for linking phytochemical variation to documented provenance [1,2,3].

Central Asia provides an especially informative setting for such studies because high floristic richness coincides with strong environmental and topographic heterogeneity. The Mountains of Central Asia are recognized for high vascular-plant diversity and endemism [1], while Kazakhstan supports more than 6000 vascular plant taxa, with the eastern and southeastern mountain systems among the principal centres of diversity [2]. Lamiaceae is prominent among regional medicinal and aromatic resources, including essential-oil-rich *Nepeta* taxa [3].

The genus *Nepeta* L. (Lamiaceae, Nepetoideae, Mentheae) comprises approximately 300 species distributed mainly across Eurasia and neighbouring temperate regions. Species of *Nepeta* are used as medicinal, aromatic, ornamental, and occasionally culinary plants and contain essential oils, iridoids, flavonoids, phenolic acids, terpenoids, and other specialized metabolites [4,5,6]. Nepetalactone and related iridoid monoterpenes are characteristic constituents of many *Nepeta* oils and contribute to aroma, ecological signalling, insect-repellent effects, and other biological activities [7,8,9,10]. Southwestern Asia and the western Himalayas are regarded as important diversification centres for the genus [11].

Ethnobotanical records provide additional context for *Nepeta* research. Across the genus, traditional applications have been reported for respiratory, digestive, nervous-system, inflammatory, aromatic, and household purposes [4,5,6,12]. Evidence specifically concerning *N. sibirica*, however, remains comparatively sparse and geographically fragmented; such records are therefore treated here as background rather than as evidence of efficacy.

Essential-oil information also remains uneven for Central Asian *Nepeta*, particularly for voucher-confirmed wild mountain populations. Regional studies have documented terpenoid-rich and, in some taxa, nepetalactone-dominant oils [13,14,15], but directly comparable data for *N. sibirica* populations from Eastern Kazakhstan are limited. This gap is important because environmental heterogeneity and population identity can contribute to variation in specialised metabolism.

Reliable botanical anchoring is essential for regional chemical comparisons. Kazakhstan studies combining population documentation with morphological, phytochemical, and bioactivity data illustrate the value of traceable plant material [16,17]. A broader methodological example shows that combining morphological characterization with multigene phylogeny can strengthen taxonomic resolution when species boundaries are uncertain [18]. Pollen micromorphology provides additional taxonomic resolution within *Nepeta* [19]. Such integrative evidence reduces the risk of assigning chemical profiles to incompletely documented material.

*N. sibirica* is a perennial Eurasian species recorded from Kazakhstan in taxonomic and herbarium sources [20,21]. It occurs on mountain slopes, in steppe meadows, along river-associated habitats, and on disturbed or fallow ground. Diagnostic characters include a perennial habit, a woody creeping underground system, quadrangular shoots, opposite leaves, and violet bilabiate flowers.

Published essential-oil profiles indicate pronounced geographic and cultivation-associated variation within *N. sibirica*. Material from the Siberian Altai was strongly nepetalactone-dominant [22], and Mongolian material was reported as a single-component nepetalactone oil [23]. In contrast, cultivated Lithuanian material showed a 1,8-cineole- and caryophyllene-oxide-rich profile [24]. These differences raise the unresolved question of whether wild Eastern Kazakhstan material is chemically closer to the nepetalactone-rich Altai–Mongolian pattern or represents a distinct regional profile.

Interpretation of nepetalactone profiles requires stereochemical caution. Conventional achiral GC–MS and retention-index matching can support compound identification but cannot by themselves establish absolute stereochemical configuration. Assignments in *N. sibirica* have been refined using chiral GC–MS and complementary stereochemical approaches [25]. In addition, iridoid production in *N. sibirica* can respond to pathway regulation and biological elicitation [26]. Variation among populations may therefore reflect interacting genetic, developmental, environmental, and biological influences rather than geography alone.

More broadly, regional studies emphasize the value of voucher-based, species-level documentation and the integration of biodiversity records with ethnobiological, chemical, and bioactivity evidence [27,28,29,30,31,32]. This methodological perspective is relevant to *N. sibirica* because taxonomically secure material and traceable provenance are prerequisites for defensible chemical and applied interpretation.

Complementary studies using field inventories, micromorphological evidence, molecular confirmation, sequence-supported occurrence documentation, and metabolite or bioactivity profiling further demonstrate the value of traceable biological material in comparative research [33,34,35,36,37,38]. These studies provide methodological context for the present work but are not treated as direct evidence for the biology or chemistry of *N. sibirica*.

The Kazakhstan Altai provides a particularly relevant regional setting. Medicinal-plant resource surveys and floristic studies have documented substantial ecological heterogeneity, population-level variation, and conservation value across the South-Western and Southern Altai [39,40,41,42,43]. Regional chemical and pharmacological studies likewise illustrate the importance of linking composition and biological activity to clearly documented provenance [44,45]. Together, these observations support population-specific interpretation of Eastern Kazakhstan *N. sibirica* rather than extrapolation from a single locality to the species as a whole.

Against this background, this study aimed to characterize a voucher-confirmed wild population of *N. sibirica* from the Altai–Tarbagatai Ridge of Eastern Kazakhstan, focusing on its botanical characteristics, essential-oil composition, and preliminary biological activity. A further aim was to assess whether the volatile profile of this population reflects broader geographic variation reported across the Eurasian range of *N. sibirica*, thereby placing the Eastern Kazakhstan material within a wider chemogeographic context.

## 2. Results

### 2.1. Taxonomic Treatment and Population Characteristics of Nepeta sibirica

**Figure 1:**
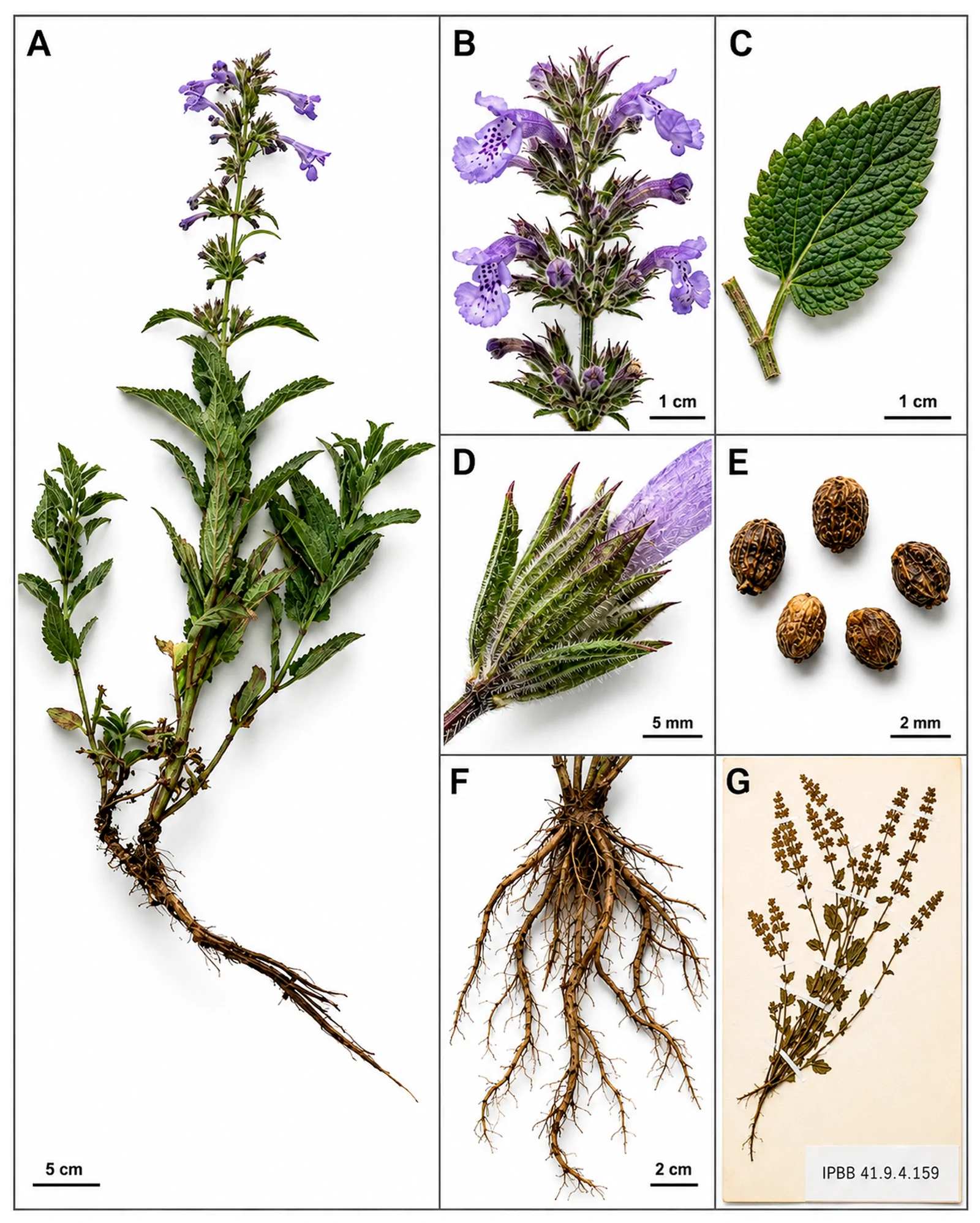
Diagnostic morphology and voucher documentation of *Nepeta sibirica* L. from Eastern Kazakhstan. (**A**) Whole flowering plant habit; (**B**) inflorescence and flowers; (**C**) leaf; (**D**) calyx and bracts; (**E**) mature nutlets; (**F**) root system; (**G**) voucher specimen deposited in the Herbarium of the Institute of Plant Biology and Biotechnology, Kazakhstan, under accession number IPBB 41.9.4.159. Scale bars: A = 5 cm; B = 1 cm; C = 1 cm; D = 5 mm; E = 2 mm; F = 2 cm.

The material was identified as *N. sibirica* from diagnostic morphology and comparison with taxonomic and herbarium references. Individuals were perennial herbs with woody creeping underground organs, erect to ascending quadrangular shoots, opposite leaves, characteristic *Nepeta* inflorescences, violet bilabiate flowers, and small mature nutlets. Figure 1A–G documents the whole-plant habit, inflorescence, leaf, calyx and bracts, nutlets, root system, and voucher specimen, thereby linking the chemical dataset to authenticated plant material.

A voucher specimen was deposited in the Herbarium of the Institute of Plant Biology and Biotechnology, Kazakhstan, under accession IPBB 41.9.4.159. This specimen provides the permanent taxonomic reference for the material used in the essential-oil analysis.

Phenology. Flowering occurs from June to September and fruiting from July to September.

Reproduction. By seed.

Population status. *N. sibirica* was common at the sampled locality and occurred in dense groups, indicating a well-established local population suitable for phytochemical sampling.

#### Morphological Description

*N. sibirica* is a perennial herb with erect stems, typically 60–100 cm tall, bearing short glandular pubescence. Leaves are oblong-lanceolate, approximately 5–15 cm long, with crenate-dentate to serrate margins, numerous punctate glands on the abaxial surface, and petioles shorter than the blades. Venation is reticulate; the veins are impressed adaxially and raised abaxially, with only weak pubescence along the veins. The inflorescence forms a relatively loose raceme of 3–8 pseudowhorls. Bracteoles are lanceolate and distinctly shorter than the calyx. The violet calyx is approximately 9.5–14 mm long, with lanceolate teeth; the teeth of the upper lip are broader and shorter than those of the lower lip. The violet corolla is 25–35 mm long and approximately two to three times the calyx length. Mature nutlets associated with the voucher material (Figure 1E) are oblong and triquetrous, brown with darker brown mottling, and bear a prominent, triangular basal hilum. Nutlet surface sculpture was not examined by stereomicroscopy, and isolated seeds were not studied separately; consequently, no additional surface-sculpture or seed characters are reported. The underground system is woody and creeping, with conspicuous lateral roots (Figure 1F). The above-ground characters, root morphology, and voucher IPBB 41.9.4.159 (Figure 1G) collectively anchor the taxonomic identity of the essential-oil sample. The principal morphological characters observed in voucher-associated material (IPBB 41.9.4.159), together with their comparison with published taxonomic and floristic descriptions, are summarized in Table 1. Habitats reported for *N. sibirica* include steppe meadows, rocky mountain slopes, river and stream banks, and fallow ground; Flora of Siberia records the species from Western and Central Siberia, Central Asia, and Mongolia [46].

### 2.2. Distribution and Ecological Context of the Sampled Population

#### Distribution in Kazakhstan Based on GBIF Records

**Figure 2:**
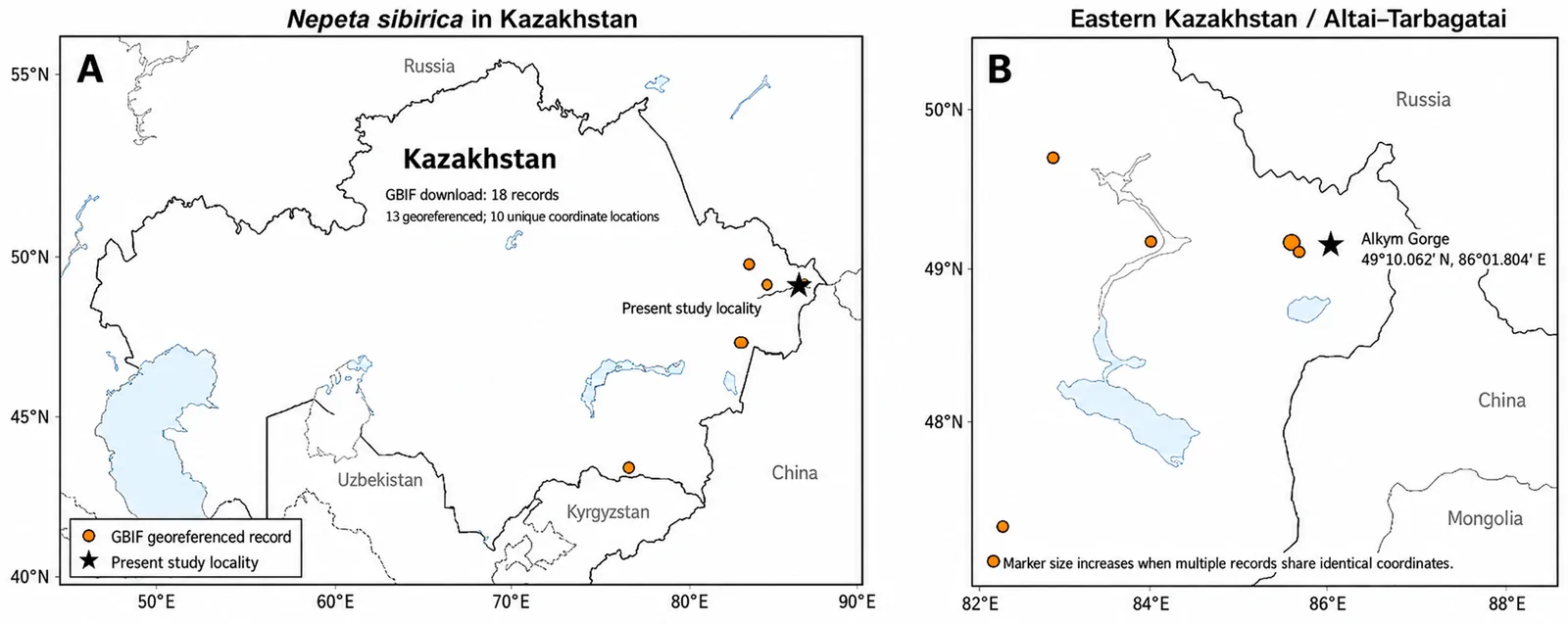
Distribution of *Nepeta sibirica* in Kazakhstan and the occurrence context of the sampled population. (**A**) Distribution of georeferenced GBIF occurrence records of *N. sibirica* across Kazakhstan. The GBIF download contained 18 Kazakhstan records, of which 13 had decimal coordinates and were used for mapping, corresponding to 10 unique coordinate locations because several records shared identical coordinate pairs. (**B**) Enlarged view of Eastern Kazakhstan and the Altai–Tarbagatai region showing the position of the present study locality in Alkym Gorge (49°10.062′ N, 86°01.804′ E; 1144 m a.s.l.) relative to nearby GBIF occurrence records. Orange circles indicate georeferenced GBIF records, with marker size increasing where multiple records share the same coordinate location; the black star indicates the present study locality. The map is intended to provide documented occurrence and collection context rather than a species-distribution model.

The GBIF occurrence dataset used in this study was obtained from a dedicated GBIF Occurrence Download (GBIF.org, 2 July 2026; https://doi.org/10.15468/dl.699bv4) [47]. The download yielded 18 occurrence records of *N. sibirica* from Kazakhstan. Of these, 13 records contained decimal latitude and longitude coordinates and were retained for mapping, representing 10 unique georeferenced localities, as several records shared identical coordinates. The georeferenced occurrences were concentrated predominantly in eastern Kazakhstan and the Altai region (Figure 2), placing the sampled population within an independently documented area of occurrence.

Sampling locality. Plant material was collected in Alkym Gorge on the Altai–Tarbagatai Ridge, Eastern Kazakhstan, at 1144 m a.s.l. (49°10.062′ N, 86°01.804′ E). The site lies within the eastern mountainous cluster represented in the GBIF dataset.

Ecological setting. The locality is part of the Southern Altai, a landscape characterised by complex relief, continental climate, and a mosaic of mountain-steppe vegetation. At the collection site, *N. sibirica* formed dense groups under mountain-steppe conditions, consistent with the range of open and semi-open habitats reported for the species.

The coincidence of the voucher-confirmed collection site with the mapped Eastern Kazakhstan occurrences provides the geographic basis for interpreting the chemical profile as belonging to a wild Altai–Tarbagatai population rather than to the species as a whole.

### 2.3. Essential Oil Yield and GC-MS Composition

Hydrodistillation of air-dried *N. sibirica* material yielded 0.3% (*w*/*w*) essential oil on a dry-weight basis. GC–MS detected and tentatively identified 62 volatile constituents accounting for 95.5% of the total composition (Table 2; Figure S1), providing broad coverage of the volatile fraction at the level supported by EI mass spectra and retention indices.

One nepetalactone peak dominated the chromatogram at 70.6%. EI mass-spectral matching and retention-index comparison supported a tentative *cis–trans*-nepetalactone assignment; however, the achiral stationary phase did not permit determination of absolute configuration or complete stereochemical identity. The principal secondary constituents were germacrene D (6.5%), *cis*-β-ocimene (2.8%), *trans*-β-farnesene (1.7%), β-caryophyllene (1.4%), β-bisabolene (1.4%), β-bourbonene (1.2%), and *trans*-β-ocimene (1.1%). Additional components included β-cyclocitral (0.7%), dihydroedulan (0.6%), caryophyllene oxide (0.5%), dihydronepetalactone (0.4%), β-elemene (0.4%), β-sesquisabinene hydrate (0.4%), 4-epi-abietol (0.4%), and several trace monoterpenes and sesquiterpenes.

The large separation between the major nepetalactone signal and all other peaks defines the sample as nepetalactone-dominant at the descriptive level. Formal chemotype designation would require replicated populations analysed under standardised conditions together with stereochemical confirmation of the major isomer.

### 2.4. Comparative Chemogeographic Profile of Nepeta sibirica Essential Oils

The Eastern Kazakhstan profile was compared with published *N. sibirica* oils from the Siberian Altai, Mongolia, and Lithuania (Table 3). Comparison was limited to studies explicitly identifying *N. sibirica* and focused on dominant constituents, oil yield when reported, and the analytical evidence used for compound identification.

The Kazakhstan sample contained 70.6% nepetalactone (tentatively assigned as *cis–trans*-nepetalactone), with germacrene D (6.5%) and *cis*-β-ocimene (2.8%) as the leading secondary constituents. Fresh flowering tops from the Siberian Altai showed a closely comparable pattern, with neoepinepetalactone at 78.8% and germacrene D at 9.4% [22]. In Mongolia, Tsuruoka et al. [23] characterised an essentially single-component oil as 4aα,7α,7aα-nepetalactone using GC–MS, optical rotation, and NMR. Cultivated Lithuanian material differed markedly, being dominated by 1,8-cineole (42.58%) and caryophyllene oxide (20.35%) [24].

These published data demonstrate substantial intraspecific chemical variation. Eastern Kazakhstan and the Siberian Altai share a nepetalactone-rich pattern, whereas the cultivated Lithuanian profile represents a distinct volatile phenotype. Because plant material, phenological stage, extraction conditions, chromatographic systems, and stereochemical resolution differed among studies, the comparison is descriptive and hypothesis-generating rather than a formal chemotype classification.

### 2.5. DPPH Radical-Scavenging Activity

**Figure 3:**
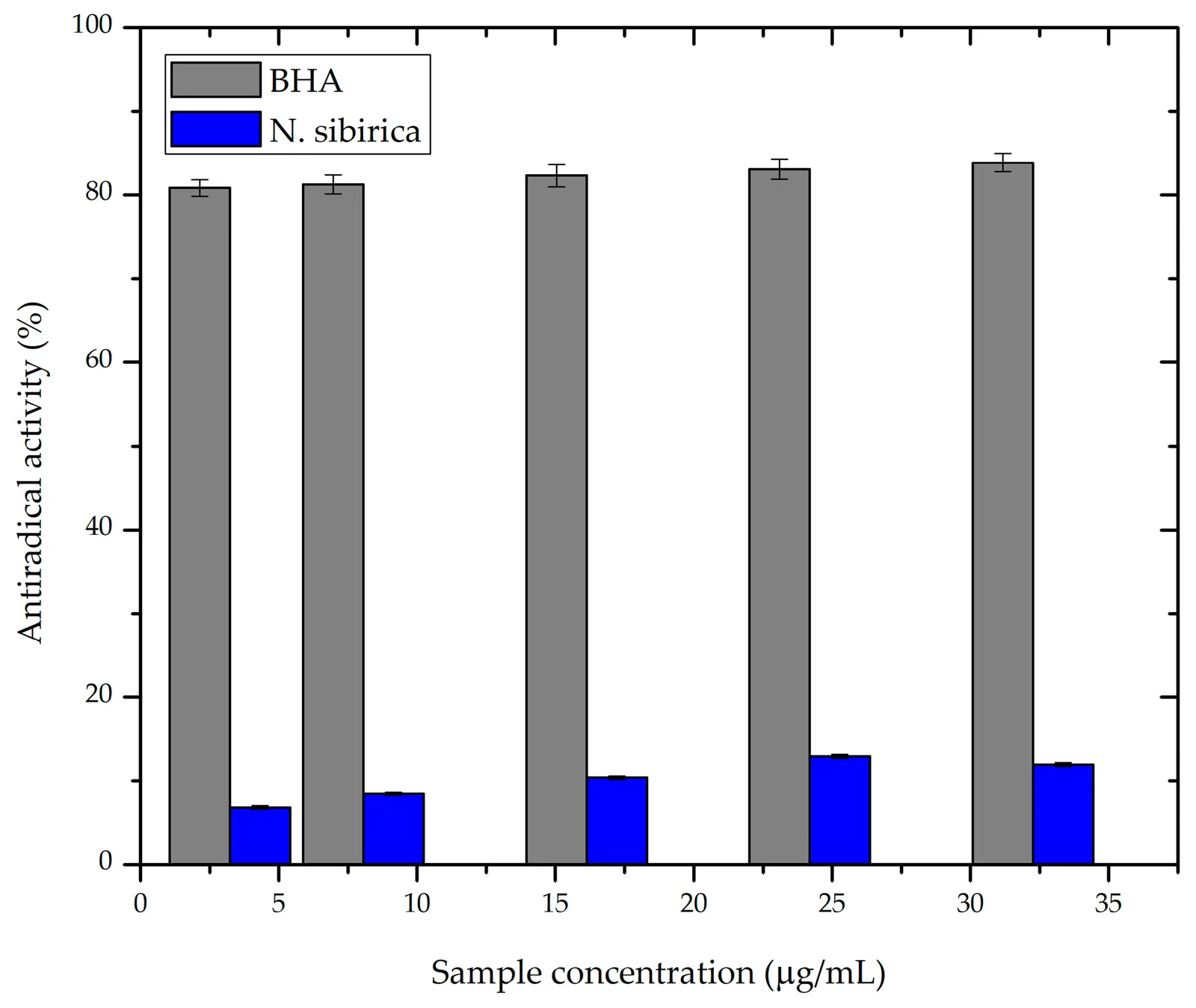
DPPH radical-scavenging activity of *Nepeta sibirica* essential oil and BHA as the reference antioxidant. Essential-oil and BHA stock solutions were prepared at nominal concentrations of 0.10, 0.25, 0.50, 0.75, and 1.00 mg/mL; after addition of 0.10 mL stock solution to 3.00 mL DPPH solution, these corresponded to final reaction concentrations of 3.23, 8.06, 16.13, 24.19, and 32.26 µg/mL, respectively, as shown on the x-axis. Data are presented as mean ± SD (n = 3).

DPPH radical-scavenging activity was evaluated for *N. sibirica* essential oil and BHA as the reference antioxidant (Figure 3; Figure S2). BHA exhibited consistently high DPPH radical-scavenging activity across the tested concentration range, with inhibition values of 80.82–83.88%.

In comparison, the *N. sibirica* essential oil showed substantially lower activity. Its DPPH inhibition increased with concentration up to a maximum of 12.88 ± 0.56% at 0.75 mg/mL stock concentration (24.19 µg/mL final concentration), followed by a slight decrease to 11.94 ± 0.48% at 1.00 mg/mL nominal stock concentration (32.26 µg/mL final concentration). As DPPH inhibition did not reach 50% at any of the tested concentrations, an IC_50_ value could not be determined within the experimental concentration range. No additional concentrations beyond those specified above were tested for IC_50_ estimation.

An exploratory PCA integrating selected GC–MS-quantified volatile constituents with DPPH-related variables is presented in Figure S3. The ordination is intended solely as a descriptive visualisation of preliminary compositional–activity patterns and should not be interpreted as evidence of statistically validated or causal relationships between individual constituents and antioxidant activity.

### 2.6. Preliminary Artemia salina Lethality Screening

*Artemia salina* screening showed low background mortality in the negative control, with approximately 96% survival (Table 4).

Exposure to *N. sibirica* essential oil produced complete mortality at 0.10, 0.05, and 0.01 mg/mL after 24 h. Because 100% mortality occurred even at the lowest concentration tested, the selected range did not resolve a dose–response relationship. No overt neurobehavioural signs were observed before death under the predefined visual criteria.

The positive control, actinomycin D, produced 100% mortality at 0.10 mg/mL and 96.2% mortality at 0.05 mg/mL based on the available larval counts.

### 2.7. Chemogeographic Synthesis of Reported Nepeta sibirica Volatile Profiles

**Figure 4:**
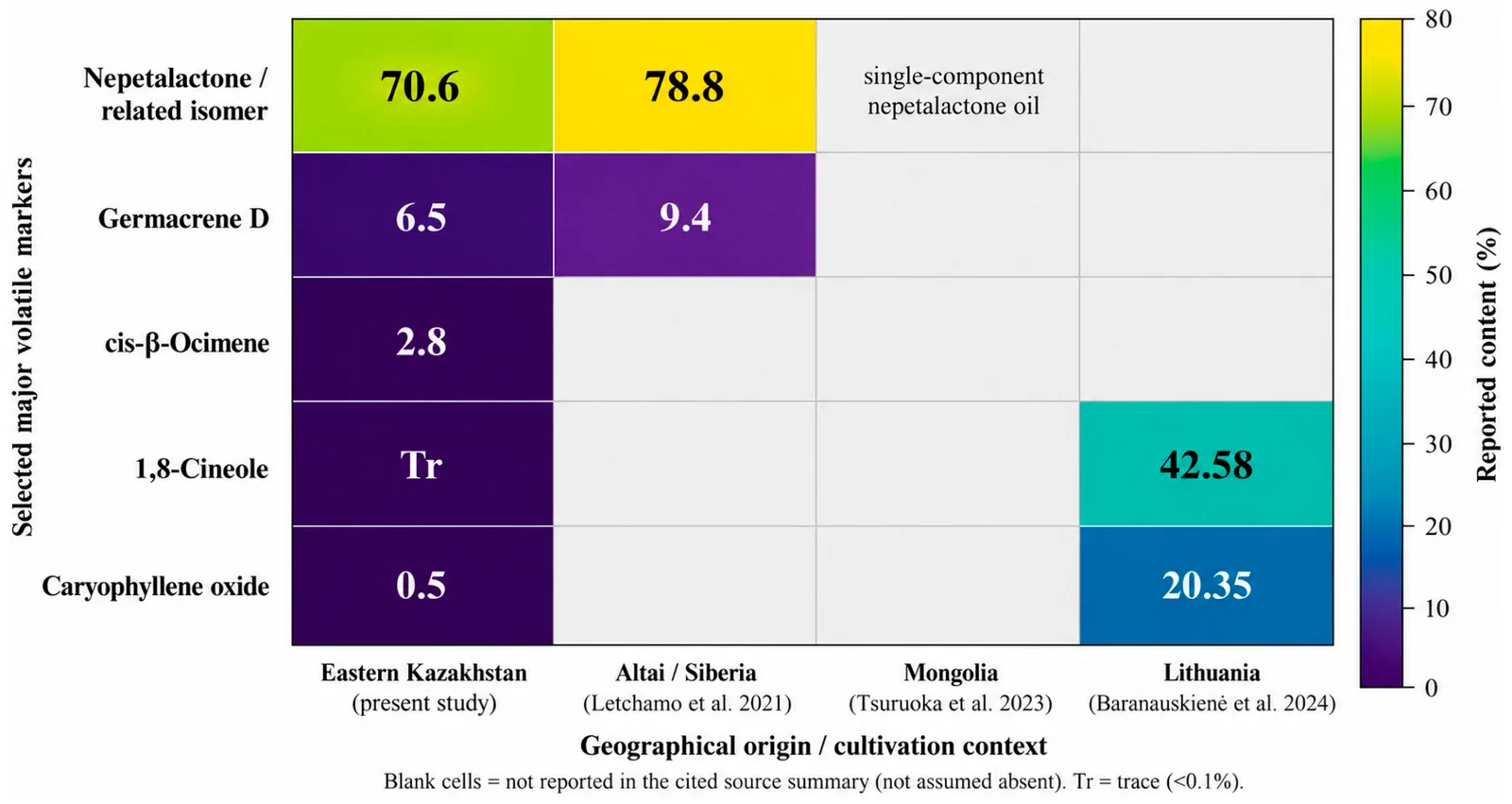
Chemogeographic comparison of selected major volatile constituents reported for *Nepeta sibirica* essential oils. Quantitative values are shown only where explicitly available for the present Eastern Kazakhstan sample, the Siberian Altai material [22], and cultivated Lithuanian material [24]. The Mongolian oil is annotated qualitatively as a single-component nepetalactone oil because Tsuruoka et al. [23] established its identity by GC–MS, optical rotation, and NMR. Blank cells indicate values not reported in the cited source summary and do not imply absence; Tr indicates a trace amount (<0.1%). Different nepetalactone labels across studies should not be assumed stereochemically equivalent.

Figure 4 condenses the major reported compositional contrasts among *N. sibirica* oils from Eastern Kazakhstan, the Siberian Altai [22], Mongolia [23], and Lithuania [24]. Only values explicitly reported in the present dataset or source summaries were plotted; missing observations were retained as blank cells rather than converted to zeros.

The matrix emphasises the shared nepetalactone dominance of Eastern Kazakhstan and the Siberian Altai, while the Lithuanian cultivated oil is distinguished by high 1,8-cineole and caryophyllene oxide. The Mongolian report is shown qualitatively because its stereochemically confirmed oil was described as single-component rather than as a full percentage profile.

Accordingly, Figure 4 is a literature-based descriptive synthesis, not an inferential multivariate analysis or a formal chemotype classification. It is intended to define a testable Altai-focused hypothesis for future replicated sampling under harmonised analytical conditions.

## 3. Discussion

Voucher-supported morphology, herbarium deposition, and accepted taxonomic records establish the identity of the Eastern Kazakhstan material as *N. sibirica* [20,21]. This taxonomic anchoring is essential because interpretation of population-level volatile chemistry depends on unambiguous species identification. Comparable Kazakhstan studies have likewise demonstrated the value of integrating morphological and phytochemical evidence [16,17], and micromorphological work within *Nepeta* provides further support for character-based taxonomic verification [19].

The spatial evidence adds a second layer of provenance. The Alkym Gorge collection falls within the eastern Kazakhstan cluster of GBIF records [47] and within the environmentally heterogeneous Southern Altai, where continental climate, complex relief, and mountain-steppe habitats may influence secondary metabolism [48,49]. The chemical profile should therefore be read as evidence from one documented wild population, not as a range-wide composition for *N. sibirica*.

This distinction is also important when discussing diversity. The study does not quantify community or species-level biodiversity; its contribution lies in documenting intraspecific phytochemical variation within a widespread medicinal and aromatic plant. Central Asian floristic inventories demonstrate high regional plant richness [1,2], while geographically structured variation has been reported in other medicinal *Nepeta* taxa [11]. Replicated sampling across the Altai–Tarbagatai, Siberian Altai, Mongolia, and additional parts of the range will be needed before geographic chemotype boundaries or broader diversity patterns can be delimited statistically.

Chemically, the Eastern Kazakhstan oil is defined by a 70.6% nepetalactone peak accompanied by substantially smaller amounts of germacrene D, *cis*-β-ocimene, *trans*-β-ocimene, *trans*-β-farnesene, β-caryophyllene, β-bourbonene, and β-bisabolene. Although EI-MS and RI data support a tentative *cis–trans*-nepetalactone assignment, stereochemical certainty is not possible from the achiral separation used here. Hernández Lozada et al. [25] showed that conventional GC assignments in *N. sibirica* can change after chiral and complementary stereochemical analysis. The strongest conclusion is therefore nepetalactone dominance; definitive isomer identification will require chiral GC–MS, co-injection with an authentic standard, or spectroscopic confirmation.

The cross-regional comparison strengthens that interpretation. Eastern Kazakhstan (70.6% nepetalactone; 6.5% germacrene D) closely approaches the Siberian Altai profile reported by Letchamo et al. [22] (78.8% neoepinepetalactone; 9.4% germacrene D). Mongolian material likewise represents an extreme nepetalactone phenotype, with 4aα,7α,7aα-nepetalactone identified as the single oil component [23]. In contrast, cultivated Lithuanian plants were dominated by 1,8-cineole and caryophyllene oxide [24]. Taken together, these observations are consistent with an Altai–Mongolian tendency towards nepetalactone-rich oils, but methodological heterogeneity and the single-population design preclude designation of a geographically fixed chemotype.

Evidence from other Central Asian *Nepeta* taxa provides useful, but not substitutive, context. East Kazakhstan *N. densiflora* has been reported with *cis–trans*-nepetalactone, *cis*-β-ocimene, germacrene D, and β-cyclocitral among its important volatiles [13], and additional regional studies document substantial variation in essential-oil composition and biological properties [14,15]. These observations reinforce the need for multi-population sampling across ecological gradients, developmental stages, and plant organs using standardised extraction and chromatographic protocols. In this sense, the current dataset contributes a well-anchored record of intraspecific chemical diversity rather than a biodiversity inventory.

Regional evidence also supports treating locality as an explicit biological variable rather than merely collection metadata. Medicinal-plant surveys in the South-Western and Southern Altai, including the Altai–Tarbagatai system, have documented substantial differences in population structure, habitat associations, and resource availability [39,40,41], while recent floristic syntheses demonstrate strong habitat heterogeneity and conservation value across the Kazakhstan Altai [42,43]. The nepetalactone-rich oil described here should therefore be regarded as a population-specific chemical phenotype that warrants replicated sampling across neighbouring ridges, seasons, and phenological stages. Kazakhstan data from *N. pannonica* [44] and regional *Rhodiola* studies [41,45] further show the value of linking chemical or biological measurements to well-documented provenance without implying that a single locality defines the species as a whole.

Mechanistic evidence offers a plausible biological basis for such variation. Popović et al. [26] showed that iridoid production in *N. sibirica* can be enhanced by transient expression of pathway-related genes and by elicitation with *Trichoderma harzianum* and *T. viride*. Nepetalactone abundance may therefore reflect regulated defence metabolism and plant–microbe interactions in addition to genetic, developmental, and abiotic influences. Resolving these contributions will require replicated field populations accompanied by environmental metadata and stereochemical resolved metabolite profiles.

The DPPH assay showed that the nepetalactone-rich oil exhibited limited radical-scavenging activity. Maximum inhibition was 12.88% at the nominal 0.75 mg/mL stock concentration (24.19 µg/mL final concentration), whereas BHA showed consistently high inhibition (>80%) across the tested concentration range. The observed weak activity is consistent with the generally weaker DPPH-scavenging capacity of volatile terpenoid-rich mixtures compared with phenolic-rich extracts. In *Nepeta* species, antioxidant activity has more frequently been associated with polar phenolic constituents, particularly rosmarinic acid [50,51,52,53,54,55]. Therefore, antioxidant activity should be considered a secondary aspect of the biological profile of the investigated essential oil rather than a major functional characteristic.

The antioxidant assessment has several limitations. First, DPPH inhibition did not reach 50% within the tested concentration range, and no additional concentrations were tested; consequently, an IC_50_ value could not be determined. Second, antioxidant activity was evaluated using a single radical-scavenging assay, which provides only a partial measure of antioxidant potential. Complementary assays based on different reaction mechanisms, including ABTS, FRAP, and CUPRAC, would provide a broader assessment. The exploratory PCA (Figure S3) is therefore retained only as a descriptive visualisation of preliminary compositional–activity patterns and should not be interpreted as evidence of statistically validated or causal relationships between individual volatile constituents and antioxidant activity.

In contrast, the *Artemia salina* assay produced a markedly different biological response, as all tested concentrations of the essential oil caused 100% mortality after 24 h. The *A. salina* lethality assay is useful as a first-pass screening approach for biologically active natural products, but the observed mortality represents a preliminary indication of biological/toxicological activity rather than a complete toxicological assessment [56,57,58]. Because neither purified nepetalactone nor individual minor constituents were tested separately, the observed lethality cannot be attributed to nepetalactone or any other specific constituent. Nevertheless, the available literature on *Nepeta* essential oils and nepetalactones supports further investigation of their potential repellent, insecticidal, antimicrobial, phytotoxic, and cytotoxic activities [6,8,9,10].

The concentration range used in *A. salina* assay was insufficient for potency estimation because complete mortality was already observed at the lowest tested concentration (0.01 mg/mL). Accordingly, lower concentrations and a broader dose–response design would be required to determine an LC_50_ value using an appropriate dose–response or probit analysis [59]. The absence of visible tremor, paralysis, loss of coordination, abnormal floating, or persistent pre-mortality immobility indicates only that no overt neurobehavioural abnormalities were observed under the experimental conditions; it does not exclude membrane-disruptive, metabolic, cytotoxic, or other underlying mechanisms.

The ethnobiological literature provides a useful boundary for applied interpretation. Central Asian studies have emphasized the value of voucher-linked integration of biodiversity, ethnomedicinal, chemical, and bioactivity evidence [27,28,29,30,31,32]. *N. sibirica* fits this broader resource framework; no new ethnobotanical interviews were conducted in the present study. Traditional or aromatic use should therefore not be conflated with demonstrated efficacy or safety, particularly in light of the strong lethality observed in the *Artemia salina* assay.

A broader methodological perspective also supports interpretation of the present dataset. Studies spanning cultivated and wild *Nepeta*, GC–MS identification, nepetalactone bioactivity, and analytical characterisation illustrate how taxonomic provenance, extraction conditions, chromatographic assignment, and functional screening jointly shape conclusions about aromatic-plant chemistry [60,61,62,63,64,65,66,67,68,69,70]. These sources are retained here to preserve the wider analytical and comparative framework of the study rather than to imply direct equivalence among experimental systems.

The functional literature further shows that volatile-plant research routinely integrates antimicrobial, anti-inflammatory, phytotoxic, antioxidant, medicinal, and cultivation-oriented evidence, while recognising that activities depend strongly on compound class, matrix, dose, and assay design [71,72,73,74,75,76,77,78,79,80]. This breadth is relevant to the present nepetalactone-rich oil because it supports a cautious transition from compositional description to targeted hypothesis testing rather than broad claims of efficacy.

Additional comparative work on essential-oil profiling, retention-index resources, insect-control applications, ecological effects, catnip chemistry, and species-specific *Nepeta* bioactivity expands the interpretive background for chemogeographic and functional variation [81,82,83,84,85,86,87,88,89,90]. Collectively, these studies reinforce the value of standardised analytical procedures and explicit reporting of missing or trace constituents when comparing populations sampled under different ecological or cultivation conditions.

Finally, cytotoxicity, toxicokinetic modelling, regional essential-oil surveys, biogeographic studies, endemic-plant chemistry, repellent activity, and comparative morphological studies provide complementary examples of how chemical, ecological, taxonomic, and safety evidence can be integrated across biological research [91,92,93,94,95,96,97,98]. Although several of these references concern systems outside *Nepeta*, they are retained as part of the manuscript’s original cross-disciplinary reference framework and support the general emphasis on reproducible identification, chemical characterization, and biologically interpretable endpoints.

Overall, the data define a voucher-confirmed Eastern Kazakhstan population with a nepetalactone-dominant volatile phenotype that is more similar to published Siberian Altai and Mongolian material than to cultivated Lithuanian plants. The most defensible applied direction is not a broad antioxidant claim, but targeted investigation of ecological and functional properties associated with nepetalactone-rich volatiles.

Future research should first address the analytical and biological limitations identified in the present study. Stereochemical confirmation of the dominant nepetalactone peak using chiral GC–MS, co-injection with authentic standards, and/or complementary spectroscopic approaches would strengthen the chemotaxonomic interpretation. Replicated sampling across regional populations, seasons, phenological stages, and ecological gradients would be necessary to distinguish population-specific variation from geographically structured chemotypes. For the biological assessment, broader concentration–response designs, including lower concentrations for the *A. salina* assay, would allow more reliable potency estimation, while complementary antioxidant assays such as ABTS, FRAP, and CUPRAC would provide a broader assessment of antioxidant behaviour.

Future phytochemical studies should also extend beyond the essential oil to different plant organs and employ extraction procedures using solvents of contrasting polarity and/or complementary extraction techniques. Combining these approaches with targeted and untargeted profiling of volatile and non-volatile constituents could help identify chemical markers associated with population, organ, developmental stage, or ecological conditions. Such chemical information should be integrated with targeted biological assays relevant to the major phytochemical classes identified. Finally, more comprehensive safety and toxicological studies using appropriate cellular and organismal models, together with mechanistic investigations, will be necessary before any broader functional or applied conclusions can be drawn from the preliminary activities observed here.

## 4. Materials and Methods

### 4.1. Study Area

The study was conducted in Eastern Kazakhstan, within the Kazakhstan Altai mountain system. The sampled population was located in the Altai–Tarbagatai Ridge, Alkym Gorge, at 1144 m a.s.l. (49°10.062′ N, 86°01.804′ E). This area belongs to the Southern Altai region, which is characterised by complex mountain relief, continental climatic conditions, and diverse vegetation types. The heterogeneity of the region, including mountain slopes, intermontane depressions, steppe–meadow habitats, and river-associated vegetation, provides suitable ecological conditions for medicinal and aromatic plants, including species of the genus *Nepeta*. The geographical and ecological context of the Southern Altai is important for interpreting possible regional variation in the accumulation of volatile secondary metabolites in wild *N. sibirica* populations. Regional resource, floristic, and physical-geography studies provide additional context for this heterogeneous mountain landscape [39,40,41,42,43,44,48,49].

### 4.2. Plant Material, Identification, and Voucher Specimen

Plant material of *N. sibirica* was collected from the Altai–Tarbagatai Ridge, Alkym Gorge, Eastern Kazakhstan, at 1144 m a.s.l. (49°10.062′ N, 86°01.804′ E). The collection was carried out by Talant Samarkhanov and Anar Myrzagaliyeva with prior permission from the relevant local executive authorities and in accordance with the Law of the Republic of Kazakhstan “On the Plant World” (No. 183-VII) [99]. The collected material consisted of aboveground plant material.

The species was identified as *N. sibirica* by Dr Anar Myrzagaliyeva based on diagnostic morphological characters and comparison with herbarium and taxonomic references. Morphological documentation included whole plant habit, stem and leaves, inflorescence and flowers, fruits/nutlets or seeds, root system, and voucher material. A voucher specimen was deposited in the Herbarium of the Institute of Plant Biology and Biotechnology, Kazakhstan, under accession number IPBB 41.9.4.159. All field sampling and experimental work involving this species were conducted in compliance with applicable institutional, national, and international guidelines and legislation.

### 4.3. GBIF Occurrence Data and Distribution Mapping

Occurrence data for *N. sibirica* were obtained from the Global Biodiversity Information Facility [47] to visualise the known distribution of the species in Kazakhstan and to place the collection locality within a broader geographical context. The GBIF download (0009522-260623161305970; accessed 2 July 2026) contained 18 occurrence records. 13 records had decimal latitude and longitude values and were used for mapping; the five records lacking coordinates were retained in Supplementary Data S1 but were not plotted. The 13 georeferenced records corresponded to 10 unique coordinate locations because several records shared identical coordinates.

The distribution map was prepared using GBIF occurrence records and the geographical coordinates of the present study locality. GBIF occurrence records were shown as circular markers, while the collection locality of the present study in the Altai–Tarbagatai Ridge, Alkym Gorge, Eastern Kazakhstan, was indicated by a star symbol. The map was used only to provide distributional and geographical context and was not interpreted as a complete species distribution model.

### 4.4. Essential Oil Isolation

The collected plant material was air-dried at room temperature under shaded and well-ventilated conditions and then shredded before essential oil isolation. Essential oil was obtained by hydrodistillation using a Clevenger-type apparatus for 2 h, following the procedure described by Yerezhepova et al. [17], with hexane used as the collection solvent. The obtained essential oil was separated and stored in sealed glass vials until GC–MS analysis and bioactivity assays. The essential oil yield was 0.3% (*w*/*w*), calculated relative to the dry weight of the plant material.

### 4.5. GC-MS Analysis of Essential Oil

The chemical composition of *N. sibirica* essential oil was determined by gas chromatography–mass spectrometry (GC–MS) using a Clarus SQ 8 gas chromatograph coupled to a mass spectrometric detector (PerkinElmer, Waltham, MA, USA). Approximately 25 mg of essential oil was placed in a 25 mL volumetric flask, dissolved in 15 mL of hexane, brought to volume, and mixed thoroughly before analysis.

GC–MS analysis was performed on a Restek Rxi^®^-1 ms capillary column (30 m × 0.25 mm i.d., 0.25 μm film thickness; Restek Corporation, Bellefonte, PA, USA). The injection volume was 1.0 μL, with a split ratio of 1:25. Helium was used as the carrier gas at a flow rate of 1.0 mL/min. The oven temperature was initially set at 40 °C and then increased at 2 °C/min to 280 °C. The injector temperature was 280 °C, and the detector temperature was 240 °C. Mass spectra were recorded in electron ionization mode at 70 eV over an *m*/*z* range of 39–500, with an acquisition time window of 4–120 min.

Relative abundances of individual constituents were calculated by peak-area normalization of the total ion chromatogram (TIC) and expressed as percentages. No compound-specific response factors were applied; the reported values therefore represent relative chromatographic abundances rather than absolute concentrations.

Compound identification was based on the combined evaluation of electron-ionization mass spectra against the NIST and Wiley spectral libraries and comparison of calculated retention indices with literature data. Retention indices were calculated relative to a homologous series of n-alkanes (C8–C40) analysed under the same chromatographic conditions. Formal compound-specific limits of detection (LoD) and quantitation (LoQ) were not established because the analysis was designed for qualitative/semi-quantitative essential-oil profiling rather than validated absolute quantification. Components present at <0.1% were reported as trace constituents; this value is a reporting threshold and should not be interpreted as an experimentally validated LoD or LoQ. A single numerical library-match score was not used as the sole criterion for identification; assignments were based on the combined spectral and retention-index evidence.

Pure analyte reference standards were not co-injected to confirm retention times or retention indices of the major constituents. The C8–C40 n-alkane series was used for chromatographic indexing. Accordingly, individual constituent assignments are reported at the level supported by EI-MS and RI evidence, and the dominant nepetalactone peak remains tentative at the stereoisomer level.

Only the Restek Rxi^®^-1 ms capillary column, based on a 100% dimethyl polysiloxane (PDMS) stationary phase, was used in the present study; alternative 5–phenyl, polar/WAX, or chiral cyclodextrin-based stationary phases were not evaluated. Because the analytical phase was achiral, definitive stereochemical assignment would require complementary analysis using a chiral GC–MS phase, co-injection with an authentic reference compound, optical rotation/circular dichroism, and/or NMR.

The GC–MS dataset used for the compositional profile was acquired in 2018. The representative chromatogram provided as Figure S1 retains the original acquisition date displayed in the analytical record; the 2018 date therefore refers to the instrumental measurement, whereas the present manuscript was prepared and submitted in 2026.

### 4.6. DPPH Radical-Scavenging Assay

The antiradical activity of *N. sibirica* essential oil was evaluated using the DPPH radical-scavenging assay, following the general principles described by Blois [50] and Brand-Williams et al. [51], with modifications adapted to essential-oil samples. Essential-oil stock solutions were prepared in hexane at concentrations of 0.10, 0.25, 0.50, 0.75, and 1.00 mg/mL. No emulsifier or additional solubilizing agent was used. For each concentration, 0.10 mL of the corresponding stock solution was mixed with 3.00 mL of DPPH solution (6 × 10^−5^ M in methanol), giving a final reaction volume of 3.10 mL. The corresponding final essential-oil concentrations were 3.23, 8.06, 16.13, 24.19, and 32.26 µg/mL, respectively.

BHA was used as a reference antioxidant and was prepared in methanol at the same stock concentrations (0.10, 0.25, 0.50, 0.75, and 1.00 mg/mL), corresponding to the same final concentrations in the reaction mixture. The negative control consisted of 3.00 mL of DPPH solution and 0.10 mL of methanol, without essential oil or BHA.

The reaction mixtures were protected from light, mixed thoroughly, and incubated in the dark for 30 min at room temperature. Absorbance was measured at 520 nm using a Cary 60 UV–Vis spectrophotometer (Agilent Technologies, Santa Clara, CA, USA). Each concentration was analysed in three independent replicates (n = 3). Antiradical activity was calculated as follows:

ARA (%) = [(A_0_ − A_t_)/A_0_] × 100

where A_0_ is the absorbance of the negative control containing DPPH solution and methanol but no essential oil or BHA, and A_t_ is the absorbance measured in the presence of the tested essential oil or BHA at the corresponding final concentration.

### 4.7. Artemia salina Lethality Assay

Preliminary toxicity of *N. sibirica* essential oil was evaluated using the *Artemia salina* lethality assay, following previously published procedures [17,18,57]. A 55 mL separatory funnel was filled with artificial seawater and supplemented with 200 mg of *A. salina* eggs. The suspension was gently aerated and maintained for 3 days to allow hatching. To generate a light gradient, one side of the funnel was covered with aluminum foil; after 5 min, nauplii that migrated toward the illuminated side were collected using a Pasteur pipette.

Approximately 20–40 nauplii were transferred into each well of a 24-well microplate containing 990 μL of artificial seawater. Baseline mortality was recorded by counting non-motile nauplii under a stereomicroscope before treatment. Each well was then dosed with 10 μL of DMSO solution containing the test sample. Actinomycin D was used as the positive control, while DMSO alone served as the negative control.

After 24 h of incubation, nauplii were examined microscopically, and the numbers of surviving and dead larvae were recorded. Samples showing strong toxicity were retested at lower concentrations of 0.10, 0.05, and 0.01 mg/mL. Nauplii were also observed for visible neurobehavioural signs, including reduced mobility, tremor-like movements, paralysis, loss of coordination, abnormal floating, and persistent immobility. Mortality was calculated using the following equation:

Mortality (%) = (dead larvae/total larvae) × 100

where dead larvae represent the number of non-viable nauplii after 24 h of exposure, and total larvae represent the total number of nauplii initially introduced into each well.

### 4.8. Statistical Analysis and Comparative Visualisation

Data from the DPPH assay were expressed as mean values with standard deviations from three independent replicates. An exploratory PCA was used only as a descriptive visualisation of selected GC–MS-quantified volatile constituents and DPPH-related variables (Figure S3). The PCA was performed using the online version of Statistics Kingdom (https://www.statskingdom.com; accessed 31 January 2026), and graphical visualisation was carried out using OriginPro 8.0.

For the chemogeographic comparison, selected major volatile constituents were compiled from the present study and published *N. sibirica* essential-oil reports [22,23,24]. Only explicitly reported quantitative values were included, while unreported values were retained as missing. The resulting matrix (Figure 4) was used for descriptive comparison of regional volatile profiles.

## 5. Conclusions

This study documents a voucher-confirmed wild population of *Nepeta sibirica* from the Altai–Tarbagatai Ridge of Eastern Kazakhstan and demonstrates a strongly nepetalactone-dominant essential-oil profile. GC–MS detected 62 volatile constituents representing 95.5% of the oil, with a dominant nepetalactone peak accounting for 70.6%, accompanied mainly by germacrene D (6.5%) and *cis*-β-ocimene (2.8%). The chemical profile supports a regional Altai-associated nepetalactone-rich pattern, although stereochemical confirmation of the dominant nepetalactone isomer remains necessary.

The essential oil exhibited limited DPPH radical-scavenging activity, whereas the *Artemia salina* assay showed 100% mortality across the tested concentration range. These latter results should be regarded as a preliminary biological/toxicological signal rather than a compound-specific or potency-defined toxicological endpoint. Future studies should therefore focus on stereochemical confirmation, replicated regional sampling, lower-dose *A. salina* assays to enable LC_50_ estimation, and targeted ecological and bioactivity investigations.

## Figures and Tables

**Table 1:** Diagnostic morphological characters of the examined *Nepeta sibirica* specimen from Eastern Kazakhstan compared with reference descriptions.

Character	Voucher-Associated Material (IPBB 41.9.4.159)	Reference Context	Interpretation/ Evidence Basis
Habit and stem	Perennial herb; erect stems, typically 60–100 cm; short glandular pubescence.	Taxonomic and floristic references support the perennial habit and diagnostic vegetative characters of *N. sibirica* [20,21,46].	Broadly concordant with species-level reference information.
Leaf morphology	Oblong-lanceolate leaves, approximately 5–15 cm; crenate-dentate to serrate margins; numerous punctate glands abaxially.	Reference descriptions support the characteristic leaf morphology of *N. sibirica* [20,46].	Broadly concordant; dimensions refer to the examined material.
Leaf venation	Reticulate; veins impressed adaxially and raised abaxially; weak pubescence along the veins.	Venation architecture is not described in sufficient detail in the cited floristic sources.	Observed directly in the examined voucher.
Inflorescence and bracteoles	Relatively loose raceme of 3–8 pseudowhorls; lanceolate bracteoles distinctly shorter than the calyx.	Floristic descriptions provide compatible inflorescence and bracteole characters [46].	Concordant within the documented species-level range.
Calyx and corolla	Calyx violet, approximately 9.5–14 mm, with lanceolate teeth; corolla violet, 25–35 mm.	Published floristic characters are compatible with the observed floral morphology [46].	Concordant within the documented species-level range.
Mature nutlets	Voucher-associated mature nutlets are oblong and triquetrous, brown with darker brown mottling, with a basal, triangular, prominent hilum.	Nutlet morphology is not described in sufficient detail in the cited floristic sources.	Observed directly in the examined voucher; surface sculpture was not assessed stereomicroscopically.
Isolated seed characters	Not examined separately from the nutlets.	Not assessed.	No separate seed-level characters are included because isolated seeds were not examined.

Note: Observations reported for the examined voucher are limited to characters directly supported by the specimen and original botanical notes. Nutlet surface sculpture was not examined stereomicroscopically, and isolated seeds were not examined separately; therefore, no additional surface-sculpture or seed-level characters are reported. Reference context follows sources cited in the manuscript [20,21,46].

**Table 2:** GC–MS composition of *Nepeta sibirica* essential oil from Eastern Kazakhstan.

Retention Time	RI	RIlit	Name of Component	Content (%)
8.281	886	893	Styrene	Tr
12.981	964	973	Sabinene	Tr
13.212	967	975	β-Pinene	Tr
13.645	974	978	1-Octen-3-ol	0.2
13.960	979	980	3-Octanone	Tr
14.269	983	991	β-Myrcene	Tr
14.665	990	996	3-Octanol	0.1
16.756	1018	1015	1-Acetylcyclohexene	Tr
16.991	1021	1028	Limonene	Tr
17.123	1023	1031	1,8-Cineole	Tr
17.860	1033	1038	*cis*-β-Ocimene	2.8
18.011	1035	1036	Benzeneacetaldehyde	Tr
18.583	1042	1048	*trans*-β-Ocimene	1.1
22.861	1098	1100	Linalool	0.1
23.224	1102	1104	2-Methylbutyl 2-methylbutanoate	Tr
23.525	1105	1107	Isopentyl isovalerate	Tr
23.694	1107	1110	2-Methylbutyl isovalerate	Tr
25.223	1124	1129	(4*E*,6*E*)-Alloocimene	0.1
29.340	1169	1177	Terpinen-4-ol	Tr
30.712	1184	1191	α-Terpineol	Tr
32.333	1202	1220	β-Cyclocitral	0.7
34.318	1226	1228	2,3,6-trimethylphenol	0.1
34.454	1227	1242	β-Citral	0.2
37.206	1261	1273	α-Citral	0.2
38.512	1277	1288	Dihydroedulan	0.6
45.790	1364	1370	Nepetalactone isomer †	70.6
46.135	1368	1378	α-Copaene	Tr
46.674	1374	1387	β-Bourbonene	1.2
47.386	1383	1385	6,8-Nonadien-2-one,6-methyl-5-(1-methylethylidene)	Tr
47.408	1383	1392	β-Elemene	0.4
48.487	1396	1401	β-Longipinene	0.1
49.129	1403	1405	Dihydronepetalactone	0.4
49.448	1407	1422	β-Caryophyllene	1.4
50.292	1416	1432	β-Copaene	0.2
51.425	1429	1445	Isogermacrene D	0.1
52.236	1438	1456	α-Humulene	0.3
52.922	1446	1458	*trans*-β-Farnesene	1.7
53.252	1450	1480	γ-Muurolene	0.1
54.441	1463	1484	Germacrene D	6.5
54.782	1467	1513	α-Curcumene	0.3
55.501	1475	1500	Bicyclogermacrene	0.1
55.978	1481	1496	α-Zingiberene	0.8
56.323	1485	1487	Guaia-1(10),11-diene	0.1
56.503	1487	1506	*cis*-α-Bisabolene	tr
56.962	1492	1510	α-Farnesene	tr
56.998	1492	1511	β-Bisabolene	1.4
57.549	1498	1517	δ-Cadinene	0.1
58.099	1506	1508	β-Sesquisabinene hydrate	0.4
61.911	1562	1586	Caryophyllene oxide	0.5
63.987	1592	1604	Humulene oxide II	0.1
64.912	1606	1608	Zingiberenol	0.3
67.597	1645	1643	τ-Cadinol	0.1
69.560	1674	1688	(1*R*,7*S*)-Germacra-4(15),5,10(14)-trien-1β-ol	0.1
70.287	1685	1688	α-Bisabolol	0.1
72.646	1719	1712	n-Pentadecanal	0.1
79.433	1818	1822	Neophytadiene	tr
81.377	1847	1849	Perhydrofarnesyl acetone	0.1
84.349	1890	1892	9,12,15-Octadecatrienal	0.3
94.316	2042	2045	8,11,13-Abietatriene	0.2
98.077	2105	2114	Phytol	tr
108.775	2385	2387	4-epi-Abietol	0.4
109.564	2440	2442	Abiet-8-en-18-oic acid	0.7
	Total			95.5

Note: RI, retention index calculated relative to a homologous series of n-alkanes (C8–C40) analysed under the same chromatographic conditions on a Restek Rxi-1 ms capillary column; RIlit, literature retention index. Relative contents (%) are TIC peak-area-normalized values and were reported without compound-specific response-factor correction. Formal compound-specific LoD and LoQ values were not determined; tr denotes a trace amount (<0.1%), which is a reporting threshold rather than a validated detection or quantitation limit. Compound identification was based on EI mass-spectral comparison with the NIST and Wiley libraries together with retention-index agreement with literature values; pure analyte reference standards were not co-injected. † The dominant nepetalactone peak was tentatively assigned as *cis–trans*-nepetalactone from EI-MS and RI evidence; because the chromatographic column was achiral, absolute configuration and full stereochemical identity were not established.

**Table 3:** Chemogeographic comparison of published *Nepeta sibirica* essential-oil profiles.

Region/Material	Yield/Analytical Basis	Dominant Constituents	Interpretation/Reference
Eastern Kazakhstan; wild aerial Material, Altai–Tarbagatai Ridge	0.3% (*w*/*w*); EI-MS + RI on achiral Rxi-1 ms	Dominant nepetalactone peak 70.6% (tentatively *cis–trans*); germacrene D 6.5%; *cis*-β-ocimene 2.8%	Nepetalactone-dominant Eastern Kazakh Altai profile; stereochemical assignment tentative. Present study.
Altai/Siberia; fresh flowering tops	1.0%; GC and GC–MS	Neoepinepetalactone 78.8%; germacrene D 9.4%	Nepetalactone-dominant Altai profile [22].
Mongolia; *N. sibirica* essential oil	NR; GC–MS + optical rotation + NMR	Single component: 4aα,7α,7aα-nepetalactone	Stereochemically confirmed single-component nepetalactone profile [23].
Lithuania; cultivated *N. sibirica* material	NR; GC–MS/FID	1,8-Cineole 42.58%; caryophyllene oxide 20.35%	Cineole/caryophyllene-oxide-rich profile [24].

Note: Only studies explicitly referring to *Nepeta sibirica* essential oil were included. NR, not reported in the cited source summary. Compound names and stereochemical assignments are reproduced according to the respective studies and should not be assumed analytically equivalent across laboratories. The comparison is descriptive because plant material, phenology, extraction conditions, chromatographic systems, and stereochemical resolution differed among studies.

**Table 4:** Preliminary toxicity of *Nepeta sibirica* essential oil in the *Artemia salina* lethality assay.

Tested Sample	Concentration (mg/mL)	Total Larvae (n)	Surviving Larvae	Dead Larvae	Survival (%)	Mortality (%)	Neurotoxicity Signs
*N. sibirica* essential oil	0.10	24	0	24	0	100	Not observed
*N. sibirica* essential oil	0.05	26	0	26	0	100	Not observed
*N. sibirica* essential oil	0.01	23	0	23	0	100	Not observed
Actinomycin D	0.10	22	0	22	0	100	Not observed
Actinomycin D	0.05	26	1	25	3.8	96.2	Not observed

Note: Survival and mortality percentages were recalculated from the numbers of total, surviving, and dead larvae. Neurotoxicity signs refer to visible neurobehavioural symptoms such as tremor-like movements, paralysis, loss of coordination, abnormal floating, or persistent immobility before death.

## Data Availability

The GBIF occurrence dataset used for the distribution analysis was downloaded from GBIF.org on 2 July 2026 (download 0009522-260623161305970). The download contained 18 occurrence records of *N. sibirica* from Kazakhstan, of which 13 contained geographical coordinates and were used for distribution mapping. All 18 records, including the five non-georeferenced records, together with mapping status, coordinates, dataset provenance, and record-level source links, are provided in Supplementary Data S1. All other data supporting the findings of this study are included in the article and Supplementary Materials.

## Supplementary Materials

The following supporting information can be downloaded at: https://www.mdpi.com/article/10.3390/plants15192992/s1, Figure S1. GC–MS chromatogram of *N. sibirica* essential oil (original analytical acquisition from 2018; acquisition date retained in the chromatogram); Figure S2. Optical density (OD) values at 520 nm measured in the DPPH assay for *N. sibirica* essential oil and BHA (butylated hydroxyanisole) at the tested concentrations (0.10, 0.25, 0.50, 0.75, and 1.00 mg/mL); Figure S3. Exploratory PCA biplot of selected GC–MS-quantified volatile constituents and DPPH-related variables. The ordination is provided as a descriptive visualization and is not intended for inferential or causal interpretation; Supplementary Data S1. Curated GBIF occurrence records of *N. sibirica* in Kazakhstan from GBIF download 0009522-260623161305970, including all 18 downloaded records, mapping status, geographical coordinates, dataset provenance, and record-level source links.
