# Supplementary material for: Wild Nepeta sibirica L. from Eastern Kazakhstan: Botanical Characterization, Essential-Oil Composition, Chemogeographic Context, and Preliminary Bioactivity
Source: Plants (Basel). 2026 Sep 30;15(19):2992. doi: 10.3390/plants15192992 (PMC13666860; doi:10.3390/plants15192992)
Supplement: Supplementary file 1 [file plants-15-02992-s001.zip › S_Material_.pdf]

SUPPLEMENTARY MATERIALS

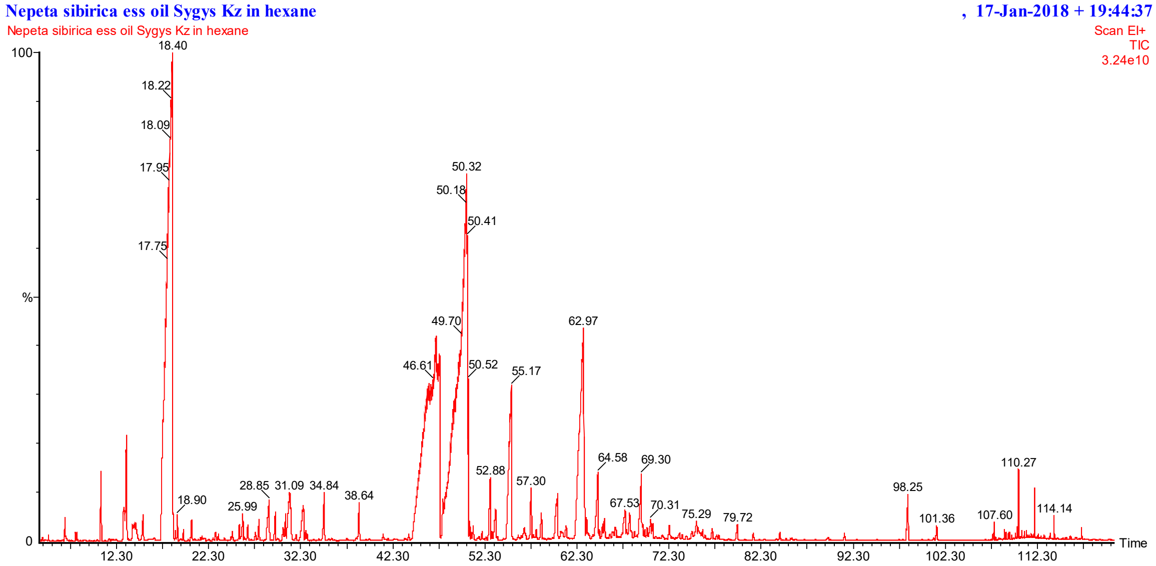

Figure S1. GC–MS chromatogram of *Nepeta sibirica* essential oil.

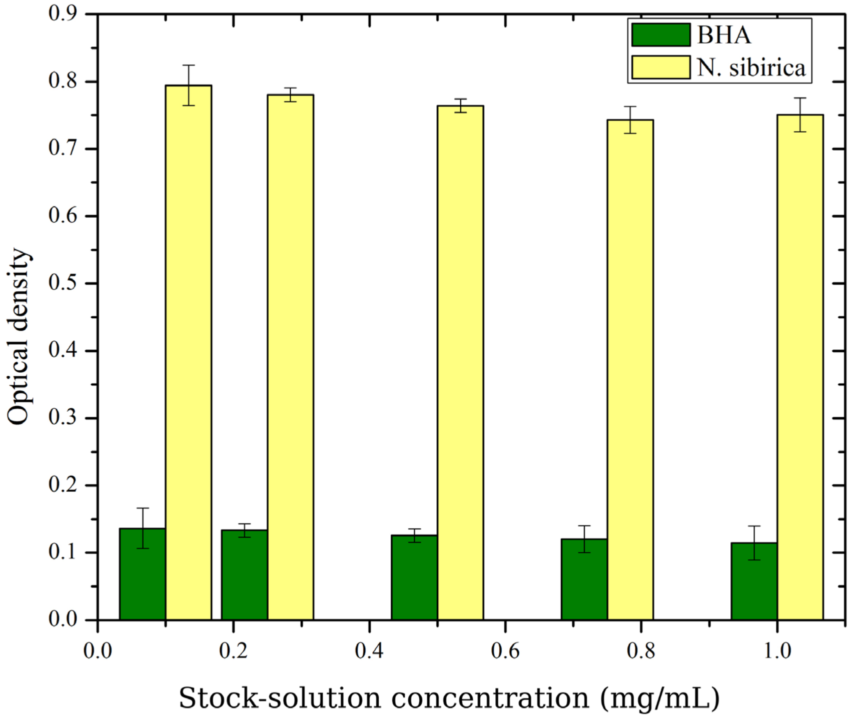

Figure S2. Optical density (OD) values at 520 nm measured in the DPPH assay for Nepeta sibirica essential oil and BHA (butylated hydroxyanisole) across nominal stock-solution concentrations of 0.10, 0.25, 0.50, 0.75, and 1.00 mg/mL. After addition of 0.10 mL stock solution to 3.00 mL DPPH solution, these corresponded to final reaction concentrations of 3.23, 8.06, 16.13, 24.19, and 32.26 μg/mL, respectively. Values are presented as mean ± SD (n = 3).

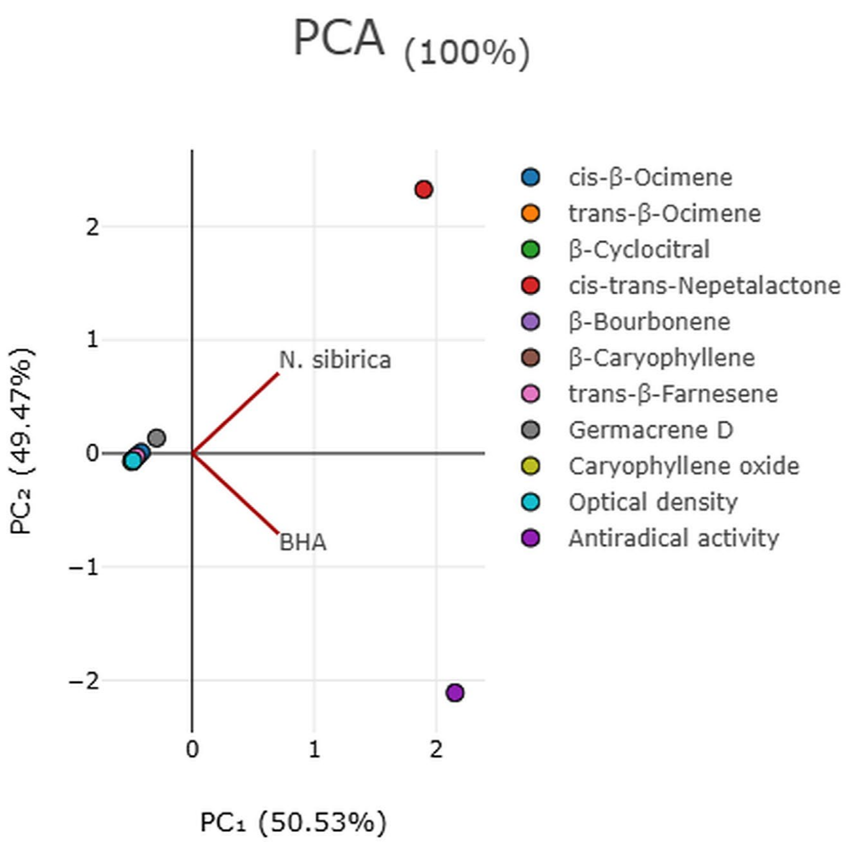

Figure S3. Exploratory PCA biplot providing a descriptive visualization of selected GC–MS-quantified volatile constituents of Nepeta sibirica essential oil and DPPH-related variables. The ordination is exploratory and should not be interpreted as evidence of statistically validated or causal relationships.

Supplementary Data S1

Supplementary_Data_S1_GBIF_Nepeta_sibirica_Kazakhstan.xlsx contains the complete 18-record GBIF download used for the Kazakhstan occurrence analysis, a curated mapping sheet, the full GBIF export, constituent dataset citations, and the present-study locality. Thirteen records had decimal coordinates and were used in Figure 2, representing 10 unique coordinate locations; five records lacked coordinates and were retained for completeness but were not plotted.

GBIF download identifier: 0009522-260623161305970; accessed 2 July 2026.
